# "Risk homeostasis"or "teachable moment"? the interaction between smoking behavior and lung cancer screening in the Mayo Lung Project

**DOI:** 10.1186/1617-9625-9-2

**Published:** 2011-01-24

**Authors:** Lu Shi, Martin Y Iguchi

**Affiliations:** 1 Department of Health Services, 650 Charles E. Young Drive S. 61-253 CHS, Los Angeles, CA 90099, USA; 2 UCLA School of Public Health, 650 Charles E. Young Drive S. 46-081C CHS Los Angeles, CA 90099 USA

## Abstract

The chest X-ray lung cancer screening program of Mayo Lung Project (MLP) yielded mixed results of improved lung case survival but no improvement in lung cancer mortality. This paper analyzes the smoking patterns of study participants in order to examine possible behavioral ramifications of periodic lung cancer screening. Using a longitudinal difference-of-difference model, we compared the smoking behavior, in terms of current smoker status among all subjects and the intensity of smoking among those continuing smokers, between those who received periodic lung cancer screening and those who received usual-care. In both arms of this lung cancer screening trial, there was a sizable decline in cigarette smoking one year after participants received baseline prevalence screening. There was no significant difference in current smoker status between the intervention group receiving periodic X-ray screening and the control group receiving usual care. While we detect that the continuing smokers in the intervention group smoked more than their counterparts in the control group, the magnitude of the difference is not sufficient to explain a substantial difference in lung cancer incidence between the two groups. Our study shows that periodic lung screening in MLP did not decrease smoking behavior beyond the observed decline following the initial prevalence screening conducted at baseline for both the intervention and control groups. Our results also indicate, paradoxically, that participants assigned to the intervention group smoked more cigarettes per day on average than those in the control group. Lung cancer screening programs need additional cessation components to sustain the abstinence effect typically observed following initial lung screening.

## Introduction

The Mayo Lung Project (MLP) was a clinical trial conducted between 1971 and 1983. It involved random assignment of male smokers to either a periodic (every 4-6 months) chest X-ray lung cancer screening program versus a usual care program that included the "treatment as usual" (TAU) recommendation to be screened yearly. The two trial arms then went through a prevalence screening, where 9,211 subjects were diagnosed as cancer-free and thus eligible for staying in the study. Those in the intervention arm then received periodic chest roentgenograms and three-day "pooled" sputum cytology studies thrice a year while lung-health questionnaires were delivered to both arms of the trial. The MLP results were mixed inasmuch as participants in the periodic screening program demonstrated longer case survival but worse lung cancer mortality as compared with those in the usual care program [1-3].

Surprisingly, no attention has been paid to the smoking behavior of MLP participants, even though smoking status has been shown to be the most important predictor of lung cancer incidence and survival. This study examines the interaction between periodic lung cancer screening and smoking behavior reported in the MLP in order to better understand the behavioral contributors to observed outcomes.

It is well established that smoking cessation could significantly reduce smokers' lung cancer mortality [4]. Further, we know that if individuals are alerted to avoidable fatal risks, they often take steps to reduce that risk [5]. Therefore, it is reasonable to infer that undergoing periodic lung cancer screening, which serves as a constant reminder of the deadly outcome of cigarette smoking, could stimulate smoking cessation among current smokers [5]. Behavioral evidence from various chronic diseases has suggested that episodic medical occasions that heighten the patient's awareness of disease risk, often called "the teachable moment"[6-8], can significantly motivate the patient to reduce or abandon the risk behavior[9-13]. For example, smokers who went through heart-related surgeries maintained an abstinence rate of 48% at 5-year follow-up, a level that no known interventions have achieved [10]. However, other than a single-arm study that showed an increase in smoking cessation efforts after people attended lung cancer screening [6], there has been insufficient documented evidence of lung cancer screening's causal impact on smoking cessation among those screened. Now that the policy debate over mandating a periodic CT screening of smokers has attracted widespread academic interest and media attention [14,15], the question of whether the screening intervention could lead to smoking cessation has become timely. This paper examines two aspects of smoking behavior observed during the MLP: the current smoking status (whether one smoked any cigarette in a given year) and the amount of cigarettes smoked per day.

## Method

The MLP tracked smoking behavior at baseline and then annually for six years [16]. We adopt a difference-of-difference longitudinal logistic model [17] to examine the screening intervention's effect on current smoker status (defined as smoking at least one cigarette vs. not smoking a single cigarette per day in the past year) and smoking intensity (defined as daily number of cigarettes consumed). We set the time period and intervention assignment as fixed effects and each participant as a random effect to examine the difference-of-difference in smoking behavior change over time across the two trial arms.

## Results

We first compared smoking behavior at baseline and the first-year follow-up, and in both trial arms we found a dramatic increase in smoking abstinence and a large decrease in number of cigarettes smoked per day. Of the 9,211 smokers enrolled in MLP, 690 quit smoking in the screening intervention group and 692 quit in the control group. On average, participants in the screening intervention group cut down on their daily smoking by 5.69 cigarettes (p < 0.001) while the control group decreased by 5.71 cigarettes (p < .001). In all, approximately 42% of participants in both groups reported a decrease in the number of cigarettes consumed. In the sixth year since MLP's baseline screening, 53.6% of the participants in the intervention arm and 57.0% of participants in the control arm reported a reduction of the number of cigarettes smoked per day compared to their baseline smoking intensity.

Results from the mixed model indicated that the screening intervention had no significant impact on current smoker status when compared to the control group, and that smoking intensity declined less in the intervention group than in the control group (Table 1). Overall, smoking intensity in the intervention group was observed to be higher by 1.90 cigarettes per day (p < 0.001). Participant age negatively predicted current smoker status (O.R. = 0.95; p < 0.001) while each additional year in age was associated with 0.51 fewer cigarettes smoked per day (p < 0.001). Years of smoking positively predicted current smoker status (O.R. = 1.03; p < 0.001) with each additional year of smoking associated with 0.15 more cigarette smoked per day (p < 0.001). Being White negatively predicts current smoker status (O.R. = 0.75; p < 0.001) and is associated with 0.82 fewer cigarettes smoked per day (p < 0.05).

**Table 1:** Difference-of-difference mixed models comparing the smoker status (1 = smoking 0 = abstinence) and smoking intensity (daily consumption of cigarettes) between the screening intervention group and the control group in Mayo Lung Project of Chest X-rays

	Current Smoker Status	Smoking Intensity
	abstinence = 0, smoker = 1 (Odds Ratios)	Linear regression coefficients
Age	0.95***	-0.51***
Years of Smoking	1.03***	0.15***
Had cancer Before	1.03	-0.24
Race (White)	0.75***	-0.82*
Screening intervention	1.03	1.90***

* *p *< .05, *** *p *< .001

## Discussion

Among the many studies that have analyzed the MLP data, this study is the first to look at the smoking behavior of the participants. The finding here that smokers in the intervention arm smoked more than those in the control group does not necessarily constitute an argument against lung cancer screening. First, it is not plausible that a difference of one or two cigarettes a day could lead to significantly different health conditions among continuing smokers, and in our study no between-group differences were noted in rates of abstinence. Secondly, we did observe a sizable decline in smoking among all participants after the prevalence lung cancer screening was administered to both groups at baseline. Since it was then that some prevention counseling was given to participants, given what we know about the strong proven effect of counseling on smoking cessation [18], this large initial decline in smoking could be viewed as evidence supporting the "teachable moment" argument. Still, the fact that participants receiving frequent screening reported significantly higher rates of cigarettes smoked per day might also indicate a paradoxical response to screening. In any case, periodic lung cancer screening appears not to improve long-term abstinence as compared with one-shot screening, a result contrary to what many would have expected but which is consistent with the results reported by Cox et al [5].

From the viewpoint of "risk compensation" (whereby people adjust their risk behavior to adapt for the changed risk), it is not surprising to see that smokers in the intervention group smoked more cigarettes per day than those in the control group. The human phenomena of behaviorally offsetting exogenous risk modification has been noted and interpreted in different disciplines like economics, psychology and risk analysis [19-22], including studies of smoking behavior [23,24]. Wilde [25] proposed a theory of "risk homeostasis" that individuals seek a stable target level of risk to bear and therefore will adjust behavior in response to exogenous changes in risk to maintain that target. Wilde [26] argues that interventions that have a direct influence on accident cost could successfully alter the target risk level, like countermeasures that increase the benefits associated with accident-free driving and the costs associated with accident involvement. In our context, going through a lung screening program with repeated negative results may conceptually reduce the expected risk of dying from smoking, both by lowering the perceived probability of having lung cancer and by increasing the expected expediency of treatment. Therefore, the expected cost of smoking for participants in the intervention group is lower than for participants in the control group. In other words, the perceived fatal risk of smoking could actually be lowered by repeated negative screening results, and thus lead to more cigarettes smoked per day. Thus, it might be worthwhile to remind smokers undergoing lung cancer screenings that repeated negative results do not imply one's immunity from tobacco-induced lung cancer. Finally, the fact that periodic lung cancer screening neither increased smoking abstinence nor decreased smoking intensity also suggests a need to add smoking cessation interventions into screening programs in order to capitalize on the "teachable moment" in later screenings, as Taylor et al [6] did in their study. It takes very little time to refer a screened smoker to existent hotline counseling services like the proven tool of Quitline [27,28].

## Competing interests

The authors declare that they have no competing interests.

## Authors' contributions

LS conceived the topic, performed the analysis and drafted the manuscript. MI provided improvement suggestions. All authors read and approved the final manuscript.
